# Lessons (Not) Learned: Chicago Death Inequities during the 1918 Influenza and COVID-19 Pandemics

**DOI:** 10.3390/ijerph20075248

**Published:** 2023-03-23

**Authors:** Ruby Mendenhall, Jong Cheol Shin, Florence Adibu, Malina Marlyn Yago, Rebecca Vandewalle, Andrew Greenlee, Diana S. Grigsby-Toussaint

**Affiliations:** 1 Department of African American Studies and Sociology, University of Illinois Urbana-Champaign, Champaign, IL 61820, USA; 2 Carle Illinois College of Medicine, University of Illinois Urbana-Champaign, Champaign, IL 61801, USA; 3 Department of Behavioral and Social Sciences, Brown University School of Public Health, Providence, RI 02912, USA; 4 Department of Community and Population Health, Lehigh University, Bethlehem, PA 18015, USA; 5 CyberGIS Center for Advanced Digital and Spatial Studies, University of Illinois Urbana-Champaign, Champaign, IL 61820, USA; 6 Department of Geography and Geographic Information Science, University of Illinois Urbana-Champaign, Champaign, IL 61820, USA; 7 Department of Urban and Regional Planning, University of Illinois Urbana-Champaign, Champaign, IL 61820, USA; 8 Department of Epidemiology, Brown University School of Public Health, Providence, RI 02912, USA; 9 Center for Health Promotion and Health Equity, Brown University School of Public Health, Providence, RI 02912, USA

**Keywords:** COVID-19, 1918 influenza, black, social death, vulnerability, pandemic, racial disparity, social determinants of health

## Abstract

During historical and contemporary crises in the U.S., Blacks and other marginalized groups experience an increased risk for adverse health, social, and economic outcomes. These outcomes are driven by structural factors, such as poverty, racial residential segregation, and racial discrimination. These factors affect communities’ exposure to risk and ability to recover from disasters, such as pandemics. This study examines whether areas where descendants of enslaved Africans and other Blacks lived in Chicago were vulnerable to excess death during the 1918 influenza pandemic and whether these disparities persisted in the same areas during the COVID-19 pandemic. To examine disparities, demographic data and influenza and pneumonia deaths were digitized from historic weekly paper maps from the week ending on 5 October 1918 to the week ending on 16 November 1918. Census tracts were labeled predominantly Black or white if the population threshold for the group in a census tract was 40% or higher for only one group. Historic neighborhood boundaries were used to aggregate census tract data. The 1918 spatial distribution of influenza and pneumonia mortality rates and cases in Chicago was then compared to the spatial distribution of COVID-19 mortality rates and cases using publicly available datasets. The results show that during the 1918 pandemic, mortality rates in white, immigrant and Black neighborhoods near industrial areas were highest. Pneumonia mortality rates in both Black and immigrant white neighborhoods near industrial areas were approximately double the rates of neighborhoods with predominantly US-born whites. Pneumonia mortality in Black and immigrant white neighborhoods, far away from industrial areas, was also higher (40% more) than in US-born white neighborhoods. Around 100 years later, COVID-19 mortality was high in areas with high concentrations of Blacks based on zip code analysis, even though the proportion of the Black population with COVID was similar or lower than other racial and immigrant groups. These findings highlight the continued cost of racial disparities in American society in the form of avoidable high rates of Black death during pandemics.

## 1. Introduction

The extraordinarily high number of COVID-19 cases and deaths have created collective grief and mourning in the U.S. and around the world. The pandemic has exacerbated critical conversations around economic and racial issues due to rates of age-adjusted mortality rates for Black people, Native Americans, and Latinx that are disproportionately higher than for white individuals [1]. In Chicago, COVID-19 has disproportionally affected multi-generational Black families and neighborhoods due to challenges with social distancing and pre-existing health conditions [2]. Furthermore, COVID-19 risk factors are also closely related to social determinants of health that have negatively impacted marginalized groups for decades: low-resource populations in dense racially segregated areas are far from jobs (e.g., job spatial-mismatch), and employment tends to be in entry-level, high-contact jobs (e.g., employment discrimination). The aforementioned circumstances have been the vehicles through which structural inequities have threatened the health and well-being of Black individuals throughout this pandemic [3].

Absent a strong sense of urgency to address anti-Black racism and its interlocking conditions (e.g., poverty, racial segregation), the next global health crisis will continue to follow a predictable pattern of avoidable excess Black deaths. W.E.B. Du Bois summarized this situation in his 1899 book, *The Philadelphia Negro*—the first sociological case study of a Black community in the U.S.:

*The most difficult social problem in the matter of Negro health is the peculiar attitude of the nation toward the well-being of the race. There have … been few other cases in the history of civilized peoples where human suffering has been viewed with such peculiar indifference*.

[4]

This study covers two of several periods that demonstrate critical connections between anti-Black racism movements and large-scale health emergencies over the past 100 years. Examples of critical connections include (1) the Civil War, emancipation and the smallpox epidemic (1862–1868) [5]; (2) the End of WWI, the 1919 race riots in 25 cities (including Chicago) [6] and the 1918 influenza pandemic; (3) the 1968 “Hong Kong” flu pandemic, the Vietnam War and the Civil Rights Movement; and (4) the Black Lives Matter Movement and the COVID-19 pandemic.

The 1918 influenza pandemic occurred during a moment of huge transformations in racial beliefs, practices, agency, and geographical segregation. In the early 1900s, many descendants of slaves were migrating to Chicago to escape the racial terror of neo-slavery in the South [7,8]. Between 1890 and 1930, the Black population in Chicago increased by 16 times to a total of 233,903 individuals [9]. In search of housing, Blacks pushed and expanded the borders of their neighborhoods into surrounding white neighborhoods, which often ignited racial violence. Between 1917 and 1920, whites bombed a Black home every 20 days (on average) [10]. In Chicago, these struggles for housing, and anti-Black racism in general, led to a five-day riot in 1919 that resulted in the deaths of 23 Blacks and 25 whites and the injury of 342 Blacks and 142 whites [11].

Intersecting with this anti-Black racism around housing was anti-Black racism in healthcare. Emma Reynolds (a young woman denied entry into a white nursing school) and Dr. Daniel Hale Williams worked together to found Provident Hospital in the South side of Chicago, which in 1891 was the first Black hospital and Black nursing school in the United States. During the 1918 pandemic, Provident Hospital was often the only hospital that treated Black individuals in Chicago [12]. In covering African Americans and the 1918 flu pandemic, Rodney Brooks writes: “Accurate numbers showing how many African Americans contracted the diseases—or succumbed to it—aren’t available; records remain scarce, since so few of those victims had contact with institutional healthcare providers or agencies” [13].

Although political, housing, and employment opportunities for Blacks have improved since 1918, we argue that the continued inextricable linkages between anti-Black racism, neighborhood segregation and access to healthcare continue to negatively affect Black life and death in Chicago. Therefore, these historical intersections should be interrogated to provide context for contemporary understandings of excess Black deaths during COVID-19 and current anti-Black racism resulting in the cry “Black Lives Matter”.

This interdisciplinary study uses innovative methods to examine whether census tracts with high concentrations of descendants of enslaved Africans and other Blacks living in Chicago experienced high levels of excess deaths during the 1918 influenza pandemic and whether similar patterns existed during the COVID-19 pandemic over 100 years later. We hypothesized that neighborhoods with higher concentrations of Black or Latinx individuals would have higher risk for the 1918 pandemic influenza and COVID-19 compared to whites.

## 2. Methods

### 2.1. Setting

The city of Chicago was selected as the geographic location for this study due its centrality in the Great Migration of Blacks from the southern to the northern United States between 1910 and 1970 [14].

### 2.2. Spatial Boundaries and Neighborhoods

To assess whether Blacks living in Chicago were vulnerable to excess death during the 1918 influenza pandemic, data from the book *A Report of an Epidemic of Influenza in Chicago Occurring during the Fall of 1918* was digitized and analyzed [15]. The report contains seven consecutive weekly maps of Chicago (from the week ending on 5 October 1918 to the week ending on 16 November 1918) that illustrate the locations of influenza deaths and pneumonia deaths, either of which may be attributed to the pandemic. Each point on the map refers to the location of a death but does not further describe the person who died.

To understand the relationship between race and space, we also identified spatial boundaries and the racial composition of 1918 Chicago neighborhoods. We used a community settlement map from Historic City–The settlement of Chicago, which was published in 1976 by the Department of Development and Planning for the city of Chicago [16]. This map shows the settlement patterns of diverse racial groups migrating to Chicago and reflects neighborhood boundaries for each race. In this article, when we refer to neighborhoods, we mean neighborhoods as defined in the Historic City map. Finally, the location of industrial areas provides a glimpse into environmental vulnerability due to residents’ proximity and potential exposure to toxic chemicals from nearby factories.

**Figure 1:**
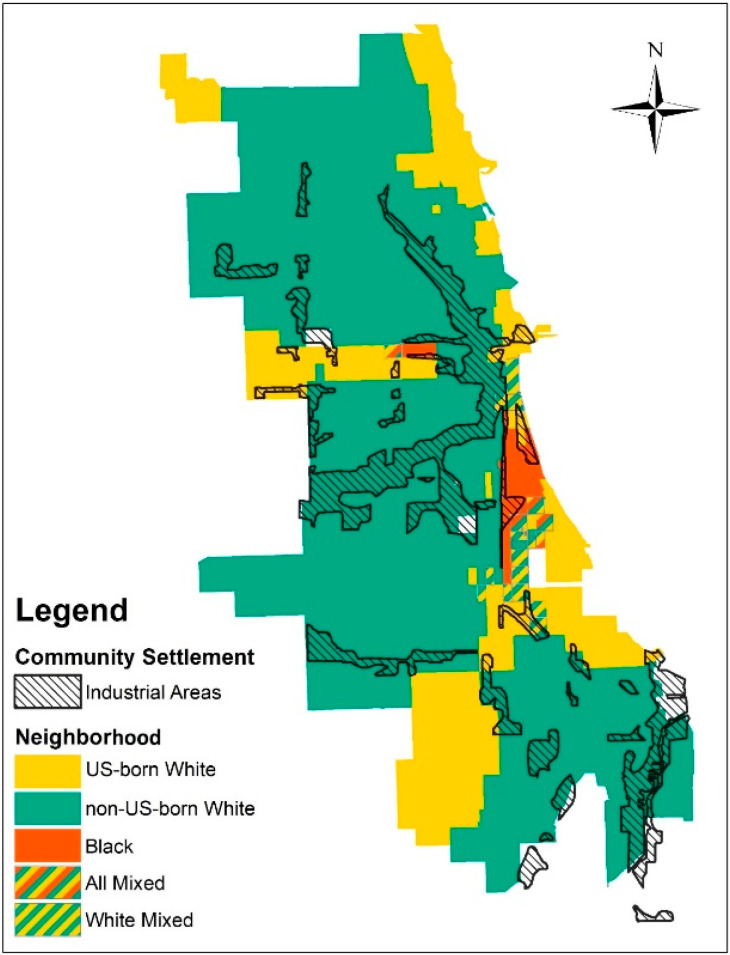
Majority race and identified industrial areas by community settlement in Chicago at the time of the 1918 influenza pandemic.

The second approach to understanding how race and space intersect is based on examining the racial composition of neighborhoods using 1920 census tracts, the smallest acquirable spatial unit. In these historic census datasets, race and nationality were originally categorized into five groups: (1) US-born White—having both parents born in the US; (2) US-born White: Foreign—having both parents foreign-born; (3) US-born White: Mix—having one parent born in the US and the other foreign-born; (4) Foreign-born White; and (5) Black. Within the category US-born White: Foreign and Foreign-born White were considered immigrants, while US-born White and US-born White: Mix were considered non-immigrants. Therefore, a total of three racial groups were considered in this analysis. The final spatial boundary of Black neighborhoods was supported via these population-based boundaries and community settlements. Figure 1 shows the spatial distribution of race and industrial areas in Chicago based on 1920 census tract data. Population data for 499 census tracts was provided by the National Historical Geographic Information System (NHGIS) [17]. For example, a census tract was classified as white if more than 40% of the residents were identified as white (this also applies to other racial groups and neighborhoods). Census tracts without any one racial group representing greater than 40% of the population were considered mixed and excluded from the analysis. To perform these analyses, the historic maps were georeferenced, i.e., digitized images of the maps were placed into a common coordinate system by matching features to known reference points. This process allows data from the historic maps to be analyzed alongside data from other historic maps as well as with modern data. Digitized point data representing influenza and pneumonia deaths were used to calculate the total number of deaths within each spatial boundary. All historic maps with geospatial information were digitized and analyzed using ArcGIS software [18].

To examine excess deaths among Blacks living in Chicago during the COVID-19 pandemic, we reviewed cumulative COVID-19 cases and deaths through 31 October 2021. The Chicago data portal, operated by the Chicago department of public health, provides publicly available COVID-19 datasets; these datasets contain general zip-code level statistics [19] and race-specific statistics for the entire city of Chicago [20]. We report race-specific cases and deaths for the entire city below, but in the initial results section, we focus on neighborhood estimates for comparability with the earlier period. Specifically, the COVID-19 datasets report deaths by zip code, which is displayed at a central location within each zip code and does not represent actual death locations. Thus, all geographic and statistical analyses for COVID-19 and identification of the majority race were performed at the zip code level. Similar to the 1918 influenza pandemic dataset, our methodological approach for COVID-19 mortality is based on estimation from the majority neighborhood in the smallest available spatial units.

## 3. Results

### 3.1. 1918 Influenza and Pneumonia

Figure 1 shows the distribution of majority race and industrial areas in Chicago at the time of the 1918 influenza pandemic. During this time, Black majority neighborhoods (Figure 1: red segments) are concentrated in the South and West Loop. Industrial areas span railroad tracks in the south and west sides of Chicago and occur along the Chicago and Calumet Rivers. Our race and space analysis shows that while most Black neighborhoods are located near industrial areas, US-born White neighborhoods (Figure 1: yellow segments) are in noncontiguous areas with minimal proximity and exposure to industrial toxins.

**Figure 2:**
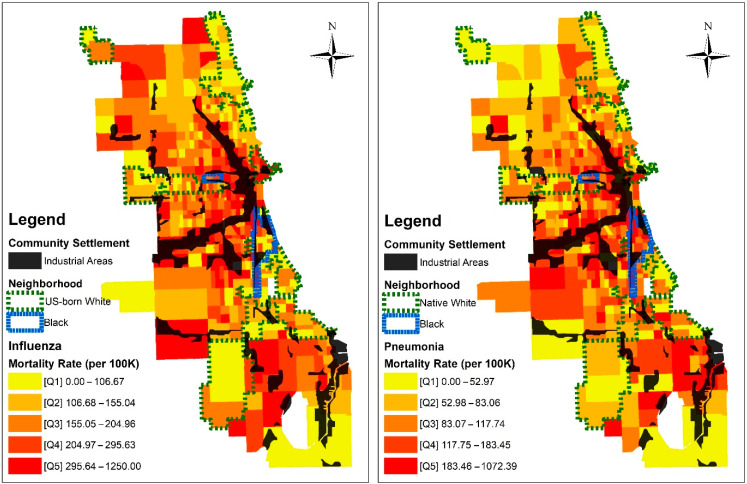
Map of the 1918 influenza and pneumonia mortality rates and census tract characteristics.

Drawing from Figure 1 and information about the racial boundaries of neighborhoods, Figure 2 illustrates the cumulative mortality rate from influenza and pneumonia by visually comparing industrial and non-industrial areas. As hypothesized, the mortality rate for influenza and pneumonia is concentrated near industrial areas, especially near Black or non-US-born white neighborhoods. In contrast, US-born White neighborhoods show evenly low mortality rates regardless of their proximity to industrial areas. (Appendix A shows the pattern of influenza and pneumonia during seven consecutive weeks in 1918.)

Table 1 provides statistics for influenza and pneumonia mortality during the 1918 pandemic. The highest mortality rates occur near industrial areas where many Black (influenza rate = 238.5 per 100,000, pneumonia rate = 168.1 per 100,000) and non-US-born White (influenza rate = 247.1 per 100,000, pneumonia rate = 155.5 per 100,000) residents lived. US-born White residents, who lived in non-industrial areas, had the lowest mortality (influenza rate = 133.7 per 100,000, pneumonia rate = 70.0 per 100,000).

### 3.2. COVID-19

**Figure 3:**
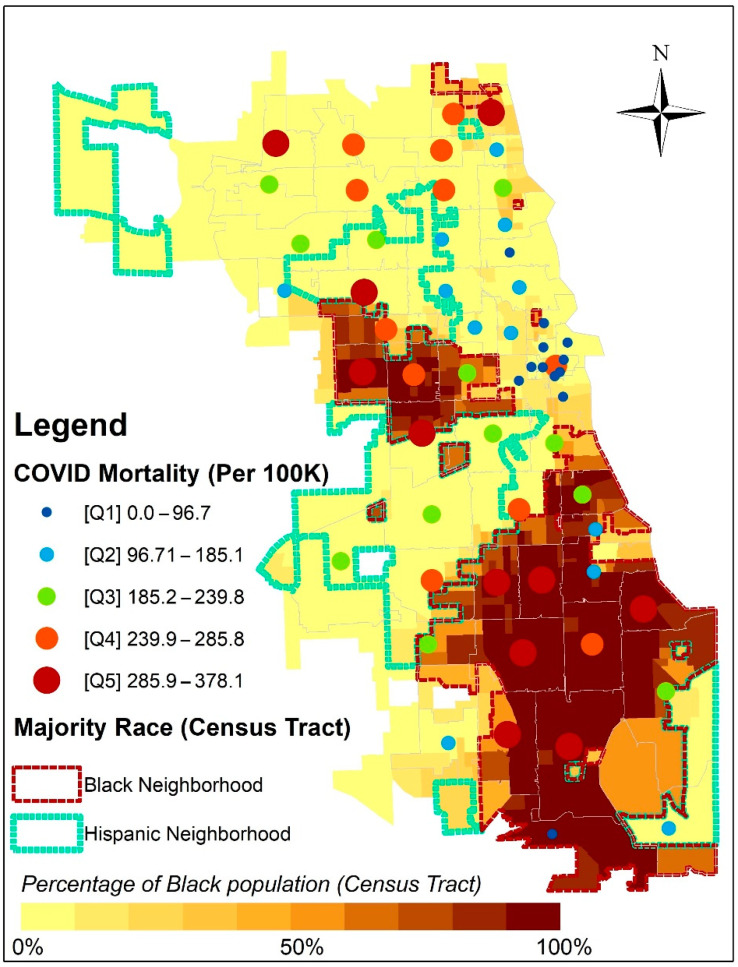
COVID-19 mortality and neighborhood characteristics by census tracts.

One hundred years later, we see similar patterns of excess deaths based on race and space. Figure 3 reveals that excess deaths from COVID-19 in Chicago are concentrated in or near Black/African American and Latinx neighborhoods. To illustrate where Black/African American and Latinx populations in Chicago predominate, census tract boundaries are used. COVID-19 mortality rates for each zip code are classified into quintiles. From the map, areas with higher levels of mortality are concentrated in the center of Black neighborhoods and become lower as one moves to the margin of Black communities where residents may have greater proximity to other racial groups and perhaps the resources (e.g., health care facilities) in the adjacent racially heterogeneous neighborhoods.

Table 2 shows COVID-19 cumulative incidence and mortality rates in Chicago using data from the majority race neighborhood estimations and recorded deaths from the City of Chicago dataset. This comparative approach to trends in cumulative incidence and mortality rates shows the feasibility of integrating historic data methods. There are some gaps between estimated and recorded numbers of COVID-19 case deaths due to the exclusion of undefined and other race, non-Latinx categories in the comparison. Although these gaps may influence an over-estimation in the cumulative incidence rate and under-estimation in the mortality rate, the comparison shows the relative consistency between estimation and actual statistics. Notably, the results show that the COVID-19 mortality rate and relative risk for the Black/African American group was the highest (actual rate = 315.0 per 100,000, RR = 2.055, 95% CI = 1.922–2.196) across all racial groups. In contrast, the estimated COVID-19 cumulative incidence rate for Black/African Americans was similar (rate = 9206 per 100,000), to whites (rate = 9192.8 per 100,000), with an insignificant relative risk (RR = 1.001, 95% CI = 0.992–1.011), with comparable results for actual cases. Our results indicate that Blacks were more than twice as likely to die from COVID-19 compared to whites using recorded case data, and just under 50% more likely using estimated data, although the cumulative incidence of the condition was similar among both groups for both case and estimated data. While Latinx people had a higher cumulative incidence of COVID-19 across estimated and case data, the estimated and recorded death rates reflect only the higher case rate, such that Latinx and white people who contracted COVID-19 had similar mortality rates. In other words, Blacks were as likely as whites to contract COVID-19 but more likely to die from the disease, while the Latinx group was more likely to contract COVID-19, and thus had higher mortality.

**Figure 4:**
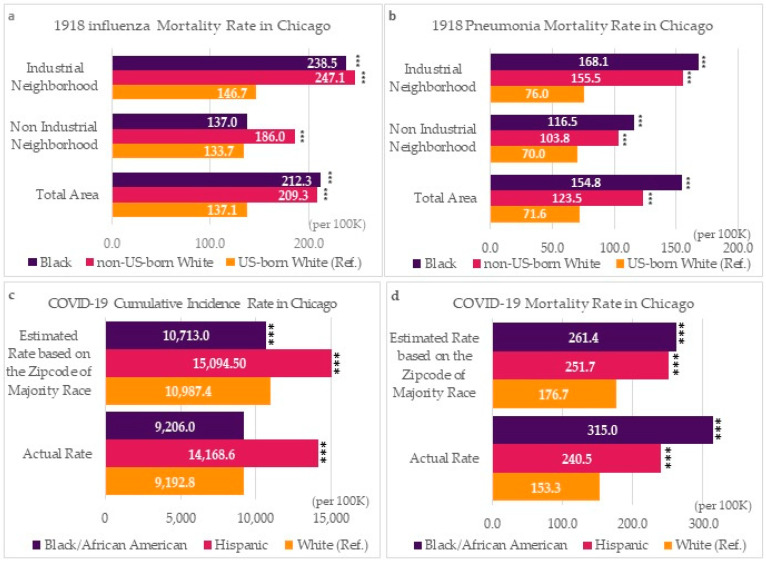
The mortality rate of the historic 1918 pandemic in Chicago. (**a**) Influenza mortality rate by race and industry area proximity, (**b**) Pneumonia mortality rate by race and industry area proximity, (**c**) COVID-19 cumulative incidence rate by race: comparison between the majority race neighbor-based estimation and true rate, and (**d**) COVID-19 mortality rate by race: comparison between the majority race neighbor-based estimation and true rate. (*** *p* < 0.001).

Comparing the historical Black excess death rate to contemporary mortality rates across the two pandemics reveals similar findings. Figure 4 compares each mortality rate by race for the 1918 influenza pandemic and the COVID-19 pandemic. Figure 4a,b show Black and African Americans had higher mortality rates for the earlier pandemic, especially for people living near industrial neighborhoods. The mortality rate for COVID-19 is also higher in Black and African American neighborhoods (Figure 4d), although the COVID-19 incidence for Blacks and African Americans was not significantly higher in both estimation and actual number (Figure 4c).

## 4. Discussion

Residents of areas inhabited predominately by Blacks and other groups of color in Chicago continue to be disproportionately impacted by the COVID-19 pandemic more than 100 years after being disproportionately impacted by the 1918 influenza pandemic. Using interdisciplinary tools from sociology, geography, urban planning, and public health, we integrate historical and contemporaneous texts, maps, and public health data to conduct a comparative analysis of the impact of racial segregation on Black/African American health in Chicago.

Our results show that areas with high numbers of Black/African American residents, or non-US-born whites that were potentially still acclimating to the socio-political and economic structures of the U.S., were at higher risk for death due to influenza and pneumonia in 1918 compared to U.S.-born whites. Industrial areas in Chicago that were characterized by low resource neighborhood conditions, including a higher prevalence of air pollution, also showed higher rates of mortality and tended to have higher numbers of Black residents and non US-born whites. Our analysis of the COVID-19 pandemic showed similar findings. Black individuals living in Chicago were twice as likely to die from COVID-19 compared to whites, and results using residential segregation patterns found similar mortality patterns for the more recent pandemic. Interestingly, Latinx groups that also comprise first, second or third generation immigrants, were also found to be at higher risk for COVID-19, similar to immigrant whites during the 1918 influenza pandemic. The higher risk for descendants of enslaved Africans and other Black individuals in Chicago should be added to extant literature on societal risks for disease among this group. Furthermore, more robust public health planning is needed to ensure that Black Chicagoans and Latinx communities do not continue to bear the disproportionate brunt of disease, particularly during times of global crisis.

### Limitations

While our study has some interesting and important findings, there are several limitations that must be addressed. First, although the total number of digitized death points for each illness was cross-referenced for all of the maps, six out of the seven maps showed that the total number of influenza and pneumonia deaths that were digitized did not equal the total number of deaths indicated in the legend, revealing a potential limitation of the digitizing process with the historical dataset. This difference is likely due to the original map resolution and its clarity on the printed page. Nearly overlapping points on the source map are hard to separate and distinguish. Additionally, some of the difference could stem from differences between the number of dots plotted and the total number of recorded deaths for the week due to plotting errors in the original map.

Second, census tracts classified as mixed (i.e., no clear racial majority) were excluded (n = 17) from our analysis. The excluded tracts accounted for approximately 2% of the population, and comparisons of pneumonia and flu mortality among racial groups in mixed neighborhoods were found to be statistically insignificant. As such, we believe the exclusion of the mixed tracts did not significantly impact our analysis.

Third, for COVID-19 data processing, the modifiable areal unit problem (MAUP) presents issues regarding zip code level data analysis. This problem arises when making comparisons between datasets with different spatial units. For example, some zip code level black neighborhoods in southern Chicago contain half of the census tract level black neighborhood and the other half of another race in a neighborhood. Although spatial matching between higher mortality and black majority neighborhoods was shown, the ambiguous boundary may not fully reflect the residential segregation by class and by racial or ethnic groups. Notwithstanding, our reliance on census tract data for determining the racial concentration of neighborhoods demonstrates the reliability of our methods.

Fourth, the finding for the COVID-19 pandemic that Black incidence was similar to the figures for whites, but with significantly higher mortality, cannot be explored in the data for the earlier pandemic. That is, it is possible that the incidence of influenza in 1918 was similar for Black and white Chicagoans, but with higher mortality for the prior group; the data simply do not allow us to explore this possibility.

## 5. Conclusions

The similarities in excess Black deaths during the 1918 influenza pandemic and COVID-19 are striking. There are several ways to learn from our findings to prevent further death inequities from occurring in the future. The higher mortality risk for Blacks observed in our work may be reflective of profiles of other health conditions in the U.S., where access to care, or insurance coverage to permit access to care, as well as health-promoting neighborhood conditions can impact the prognosis of disease in divergent ways for minoritized groups compared to whites. Consequently, the current healthcare landscape calls for interdisciplinary approaches with intersectional interventions, as coined by legal scholar Kimberlee Crenshaw [21], to create wellness care and reconfigure how we understand social determinants of health. We encourage scholars in disciplines such as critical geography [22,23] to use spatial analytics to interrogate areas with marginalized people, identify points of intervention at structural and system levels, and bring to light disparate patterns and effects of how people access and receive care during crises. Moreover, scholars from sociology and public health could argue for systemic changes in policies to make communities of color less vulnerable during pandemics.

The unique methodological approach of this study suggests ways to promote equity and health justice for Black individuals who have limited resources during global pandemics. Elimination of health disparities will take more resources than it has taken to eliminate various childhood diseases. Yet one first step could involve an interdisciplinary approach that includes human developmentalists, physicians, historians, sociologists, urban planners, public health scholars, government officials, community health workers, citizen/community scientists and community members. Such a concentration of scholarship, interventions and lived experiences could, we believe, through substantial improvements in knowledge and collaboration with policy makers, increase health and wellness in disadvantaged communities.

## Figures and Tables

**Table 1:** The estimated number of Chicago influenza and pneumonia deaths in 1918 by majority race and residential proximity to industrial areas.

	Majority Race	Total Population	Influenza Deaths			Pneumonia Deaths		
		n	n	RR	95% CI	n	RR	95% CI
Total	US-born White	564,479	774	1.000		404	1.000	
	Non-US-born White	1,899,549	3975	1.526	1.413–1.648	2346	1.726	1.553–1.918
	Black	113,058	240	1.548	1.34–1.789	175	2.163	1.811–2.582
Non-Industrial Area	US-born White	415,889	556	1.000		291	1.000	
	Non-US-born White	1,175,449	2186	1.391	1.267–1.527	1220	1.483	1.305–1.686
	Black	29,194	40	1.025	0.744–1.412	34	1.664	1.167–2.374
Industrial Area	US-born White	148,590	218	1.000		113	1.000	
	Non-US-born White	724,100	1789	1.684	1.463–1.938	1126	2.045	1.685–2.481
	Black	83,864	200	1.626	1.342–1.969	141	2.211	1.726–2.831

**Table 2:** The comparison between majority-race based estimation and actual number and rates for cumulative COVID-19 cases and deaths in Chicago between 1 March 2020, and 31 October 2021.

Majority Race	Estimated COVID-19 Cases				Actual COVID-19 Cases			
	N	Rate	RR	95% CI	N	Rate	RR	95% CI
Black/African American	86,842	10,713.0	0.975	0.967–0.983	72,502	9206.0	1.001	0.992–1.011
Latinx	100,042	15,094.5	1.37	1.36–1.38	116,114	14,168.6	1.541	1.528–1.554
White	127,283	10,987.4	1		79,391	9192.8	1	
Total	324,565	11,935.4			268,007	9758.5		
	Estimated COVID-19 Death				Actual COVID-19 Death			
Black/African American	2119	261.4	1.479	1.392–1.572	2481	315.0	2.055	1.922–2.196
Latinx	1668	251.7	1.424	1.335–1.519	1971	240.5	1.569	1.463–1.682
White	2047	176.7	1		1324	153.3	1	
Total	5996	220.5			5776	210.3		

Estimated COVID-19 cases and deaths were calculated via majority racial group analysis at the Zip-code level; while actual cases and deaths are from data from the entire city of Chicago.

## Data Availability

Please contact the corresponding author for data availability.

## Appendix A

This appendix contains weekly maps for the 1918 Chicago influenza pandemic that were used in the analysis.
